# Neural Activity and Decoding of Action Observation Using Combined EEG and fNIRS Measurement

**DOI:** 10.3389/fnhum.2019.00357

**Published:** 2019-10-15

**Authors:** Sheng Ge, Peng Wang, Hui Liu, Pan Lin, Junfeng Gao, Ruimin Wang, Keiji Iramina, Quan Zhang, Wenming Zheng

**Affiliations:** ^1^Key Laboratory of Child Development and Learning Science of Ministry of Education, School of Biological Science and Medical Engineering, Southeast University, Nanjing, China; ^2^Department of Psychology and Cognition and Human Behavior Key Laboratory of Hunan Province, Hunan Normal University, Changsha, China; ^3^College of Biomedical Engineering, South-Central University for Nationalities, Wuhan, China; ^4^Department of Graduate School of Systems Life Sciences, Kyushu University, Fukuoka, Japan; ^5^Neural Systems Group, Massachusetts General Hospital, Harvard Medical School, Charlestown, MA, United States

**Keywords:** action observation, mirror neuron system, theory of mind, complex brain network, EEG, fNIRS

## Abstract

In a social world, observing the actions of others is fundamental to understanding what they are doing, as well as their intentions and feelings. Studies of the neural basis and decoding of action observation are important for understanding action-related processes and have implications for cognitive, social neuroscience, and human-machine interaction (HMI). In the current study, we first investigated temporal-spatial dynamics during action observation using a combined 64-channel electroencephalography (EEG) and 48-channel functional near-infrared spectroscopy (fNIRS) system. We measured brain activation while 16 healthy participants observed three action tasks: (1) grasping a cup with the intention of drinking; (2) grasping a cup with the intention of moving it; and (3) touching a cup with an unclear intention. The EEG and fNIRS source analysis results revealed the dynamic involvement of both the mirror neuron system (MNS) and the theory of mind (ToM)/mentalizing network during action observation. The source analysis results suggested that the extent to which these two systems were engaged was determined by the clarity of the intention of the observed action. Based on the difference in neural activity observed among different action-observation tasks in the first experiment, we conducted a second experiment to classify the neural processes underlying action observation using a feature classification method. We constructed complex brain networks based on the EEG and fNIRS data. Fusing features from both EEG and fNIRS complex brain networks resulted in a classification accuracy of 72.7% for the three action observation tasks. This study provides a theoretical and empirical basis for elucidating the neural mechanisms of action observation and intention understanding, and a feasible method for decoding the underlying neural processes.

## Introduction

Action observation is a cognitive process enabling understanding, choosing and imitating the form and motion of an action by observing the actions of another person (Lee et al., 2012). Observing the behavior of others and understanding their intentions is an essential component of social behavior. Action understanding and imitation of others’ actions may help an observer understand the intention and emotional state of the agent involved (Libero et al., 2014). Previous studies have indicated that action observation can improve motor performance (Gatti et al., 2013) and motor skill learning (Kim et al., 2017). Moreover, as a safe and easy therapy for clinical rehabilitation and treatment of stroke, Parkinson’s disease and autism spectrum disorder, observing the actions of others has been reported to improve motor function (Harmsen et al., 2015; Caligiore et al., 2017) and facilitation of social interaction (Perkins et al., 2015). Observation and understanding the intention of action is a fundamental requirement for human-machine interaction (HMI). In HMI scenarios, action intention understanding between humans and machines is the basis of interaction. During these interactions, it is important to enable the machine to understand the human’s action intention (Casalino et al., 2018). Previous studies have made substantial progress toward enabling machines to understand the action intention of humans (Hernandez et al., 2014; Foster et al., 2017; Bandara et al., 2018). Meanwhile, it is also crucial to construct a feedback route from the human to the machine, to let the machine know that the human has understood the intention of the machine’s action during the interaction. For example, in a home care situation with HMI, a robot may have the intention to feed a user, while the user may not notice the action of the robot. In such a case, the robot should stop the action immediately to avoid a dangerous situation. However, research on this topic is currently lacking. Therefore, in the current study, we attempted to use brain signals as feedback signals for the machine, to make the machine aware of the human’s process of intention recognition.

The mirror neuron system (MNS) is believed to underlie the human ability to understand others’ actions and intentions during action observation, *via* a direct-matching process (Rizzolatti and Craighero, 2004; Kanakogi and Itakura, 2010), by which visuomotor information is transformed into motor knowledge (Jeon and Lee, 2018). The MNS provides a neural basis for recognizing the observed action. Consequently, action recognition involves the recognition of the goal of an action (i.e., action understanding; Rizzolatti et al., 2001; Iacoboni et al., 2005). The MNS responds maximally when observing object-directed interactions of a hand with an object (Rizzolatti et al., 2001) and hand-object interaction is a necessary condition to trigger the MNS (Umiltà et al., 2001). Intention understanding involves the integration of representations of the meaning and intent of the action based on hand-object interaction (Ortigue et al., 2009). The MNS mainly includes the premotor cortex (PMC), inferior frontal gyrus (IFG), superior parietal lobule (SPL), and rostral inferior parietal lobule (IPL; Molenberghs et al., 2012; Jeon and Lee, 2018).

Once others’ actions are mapped onto the observer’s own motor representation of the same action, the observer can understand the actions and predict the relationships between external states of affairs and internal states of mind, which leads to the activation of “theory of mind” (ToM; also referred to as mentalizing or mental state reasoning; Rizzolatti et al., 2001; Jeon and Lee, 2018). Unlike action observation, mental state reasoning requires high-level cognitive and attentional resources (Lin et al., 2010) and is believed to be unique to humans (Call and Tomasello, 2008). Widely distributed neural networks have been consistently implicated in ToM, including the temporoparietal junction (TPJ), superior temporal sulcus (STS), posterior cingulate cortex/precuneus (PCC/PC), medial prefrontal cortex (MPFC), and anterior temporal lobes (ATL; Rilling et al., 2004; Yang et al., 2015).

As a classical neural imaging methodology, electroencephalography (EEG) provides a measure of the electrical potentials generated by cortical postsynaptic currents. EEG not only reflects the momentary activity of a population located near the recording electrodes but also distal populations *via* the volume conduction effect (He et al., 2011). EEG has the advantage of low-cost, safety, portability, and high temporal resolution, but is limited by electromagnetic and motion interference. Functional near-infrared spectroscopy (fNIRS) is an emerging non-invasive brain-imaging methodology using near-infrared light to monitor changes in the concentration of oxyhemoglobin (HbO), deoxyhemoglobin (HbR) and total hemoglobin (HbT = HbO + HbR) in the superficial layers of the cerebral cortex beneath a pair of source and detector optodes. Because of its low cost, safety, portability, and acceptable spatial resolution, fNIRS is increasingly used in research and clinical applications (Boas et al., 2014; Kamran et al., 2016). fNIRS relies on the relationship between local neural activity and changes in regional cerebral blood flow (rCBF) affecting oxygenation and hemoglobin content in blood vessels; local cortical activation causes an increase in HbO and HbT, with a corresponding decrease in HbR (Villringer and Chance, 1997). Compared with fMRI, fNIRS has important advantages for measurement in real-world situations, including a relative lack of constraints of the experimental environment, and the robustness of the signal against motion artifacts. These features enable fNIRS to expand the potential applications of measurement in real-world environments (Balardin et al., 2017).

EEG and fNIRS differ markedly in terms of the underlying imaging principles and physiological concepts. Because EEG and fNIRS each have specific limitations (e.g., low spatial resolution for EEG, and low temporal resolution for fNIRS), the advantages of combining EEG and fNIRS measurements could provide an approach for overcoming the limitations of each method. EEG-fNIRS measurement acquires brain activation from different physiological signals, which could increase data quality and quantity. Moreover, bimodal EEG-fNIRS could provide additional information about neurovascular coupling (NVC), which is the cascade of processes by which neural activity modulates local cerebral hemodynamic properties (Keles et al., 2016). EEG electrodes and fNIRS optodes have good adaptability in terms of spatial configuration. Finally, many studies have found that bimodal EEG-fNIRS signals provide more feature information than single-mode EEG or fNIRS systems, which can significantly improve classification accuracy (Hong and Khan, 2017; Khan and Hong, 2017). For these reasons, bimodal EEG-fNIRS measurement has substantial potential in research and practical applications (Fazli et al., 2012; Ahn and Jun, 2017; Berger et al., 2018). Because action observation is a dynamic and complex spatiotemporal process (Gardner et al., 2015; Ge et al., 2017), bimodal EEG-fNIRS measurement has important advantages for exploring its dynamic processes in terms of both temporal and spatial characteristics. To the best of our knowledge, aside from previous studies in our laboratory (Zhang et al., 2015; Ge et al., 2017), no action observation studies using EEG-fNIRS bimodal measurement have been reported.

Recently, complex brain network-based graph theoretical analysis has become a powerful and popular approach for analyzing brain imaging data (Yu et al., 2018). Complex brain networks can reveal the mechanisms and characteristics of brain structure and function that cannot be discovered by past analytical methods, such as modularity, hierarchy, centrality, and the distribution of network hubs (Bullmore and Sporns, 2009). The complex brain network is a powerful approach to identify the similarities and differences of brain activation in many applications, such as brain-computer interface classification (Zhang et al., 2016), mental illness diagnosis (Fang et al., 2017; Shon et al., 2018), fatigue detection (Han et al., 2019), and emotional cognitive classification (Liang et al., 2018). However, to date, few studies have used complex brain networks to classify action observation and understanding. Consequently, exploring the whole-brain complex brain network patterns during the observation and understanding of different action intentions would be meaningful.

The current study sought to investigate the neural basis of action observation and to classify the action observation process based on brain signals. To this end, we first used bimodal EEG-fNIRS measurement to investigate temporal-spatial dynamics during action observation. We then constructed complex brain networks based on EEG and fNIRS data to classify the brain activity corresponding to action observation with different intentions. We expected that the brain activation analysis method based on EEG-fNIRS bimodal signals in the current study would extend understanding of the spatiotemporal features of the neural mechanisms underlying action intention understanding. In addition, the intention classification method in the current study could provide a new research direction for human-computer interaction research.

## Materials and Methods

### Participants

Sixteen healthy adults [six females and 10 males; mean age = 24.1 years, standard deviation (SD) = 1.3, range 22–26 years] participated in the study. None of the participants reported a history of neurological conditions or psychosis, or used medication. All participants had normal or corrected-to-normal vision and were confirmed to be right-handed using the Edinburgh Handedness Inventory. All participants provided written informed consent in accordance with the Declaration of Helsinki (World Medical Association, 2013) before enrolment in the study, which was approved by The Ethics Committee of Affiliated Zhongda Hospital, Southeast University (2016ZDSYLL002.0 and 2016ZDSYLL002-Y01). Each participant received 200 RMB for participating, after the experiment.

Before recording, participants were informed that some of the hand-cup interaction stimuli without context may be associated with the following intentions: (1) grasping a cup with the intention of drinking; (2) grasping a cup with the intention of moving it; and (3) touching a cup with an unclear intention. All participants were familiarized with each of the actions in a 3.2 min training session. After the session, participants were debriefed to ensure they understood the experimental instructions and correctly understood the action intentions shown. During EEG-fNIRS measurement, participants received clear instructions to carefully observe the three different kinds of hand-object interaction stimuli and attempt to understand the intention behind the stimuli. There is no behavioral response required from participants. To avoid eye and muscle movement interference, participants were asked to look at the cross at the center of screen and to make no verbal responses throughout the experiment.

### Experimental Procedure

**Figure 1:**
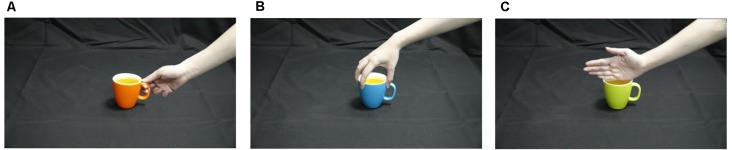
Three kinds of hand-cup interaction stimuli corresponding to different potential intentions. **(A)** a right hand grasping the handle of the cup with the intention to drink from it (Sd); **(B)** a right hand grasping the rim of a cup with the intention to move it (Sm); **(C)** a right hand touching the rim of a cup without a clear intention (Su).

**Figure 2:**
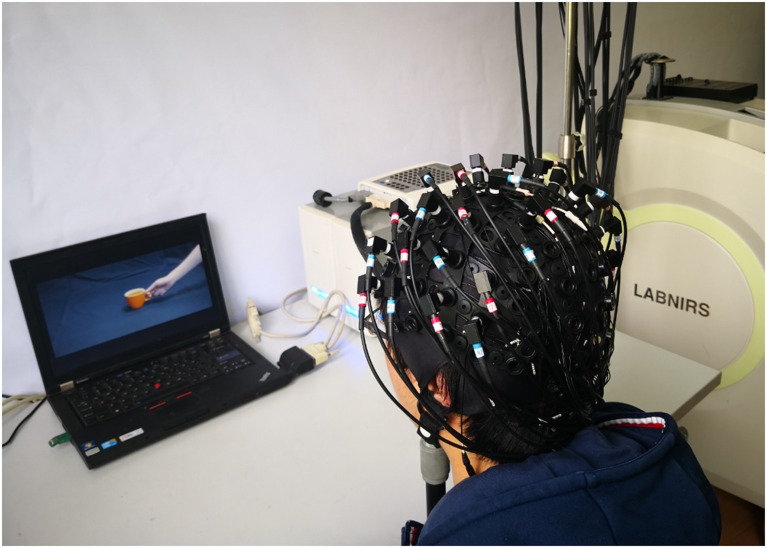
Experimental environment.

There were three kinds of hand-cup interaction stimuli (Figure 1, partially referring to Ortigue et al., 2010) corresponding to different potential intentions: (a) A right hand grasping the handle of the cup with the intention to drink from it (Sd); (b) a right hand grasping the rim of a cup with the intention to move it (Sm); and (c) a right hand touching the rim of a cup without a clear intention (Su). Participants were seated in a comfortable chair in a dark shielded room with their heads placed on a chin-rest (Figure 2). The stimuli were displayed on a computer monitor 80 cm away from the participants. The size of the stimuli was H: 28 cm (19.85° of visual angle) × V: 16 cm (11.42° of visual angle).

**Figure 3:**
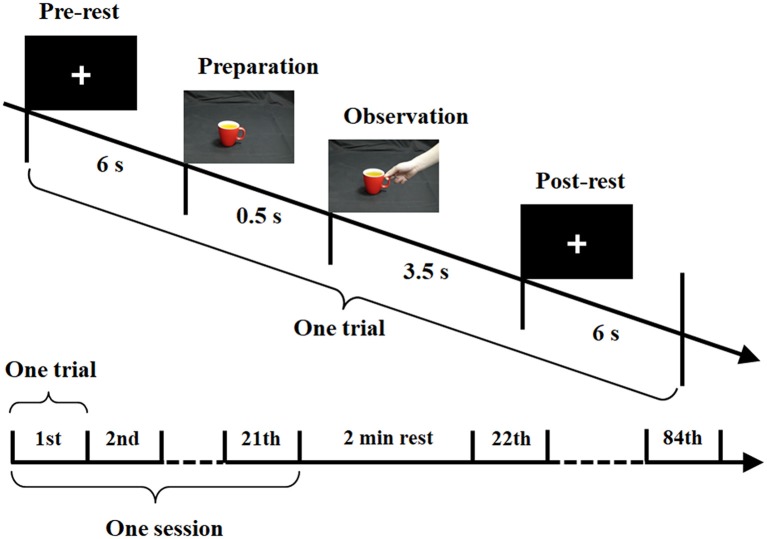
Experimental paradigm for bimodal electroencephalography (EEG)-functional near-infrared spectroscopy (fNIRS) measurement.

Figure 3 shows a schematic diagram of the bimodal EEG-fNIRS measurement procedure used in the current study. The visual stimuli were programmed in E-Prime (Version 2.0, Psychology Software Tools Inc., Sharpsburg, PA, USA). Every trial included the following sequence. First, participants underwent a pre-rest period in which a fixation cross was presented for 6 s. This was followed by a preparation period, in which a cup appeared as a cue on the screen for 0.5 s, notifying the participant to prepare for the upcoming observation period. Then, in the observation period, a hand-cup interaction stimulus was presented for 3.5 s. Since the temporal interval between the preparation and observation periods was very short, a continuous image sequence could generate the perception of an action (Brown et al., 2010; Ortigue et al., 2010). During the observation period, participants were instructed to try to understand the intention corresponding to each observed stimulus. For convenience, the starting moment of observation period was defined as 0 time point. Finally, there was a post-rest period, in which participants rested for 6 s. The post-rest and pre-rest of two subsequent trials were used as the baseline period. To avoid an adaptation effect, the three hand-cup interaction conditions were presented in a random sequence. The color of the cup alternated randomly among seven colors, and each color was shown four times in the whole experiment. The whole experiment for each participant consisted of a total of 84 trials (three hand-cup interaction conditions × seven colors × four times) and divided into four equal sessions lasting for 28.4 min. Each session was composed of 21 trials, with a 2-min rest between sessions.

### Data Acquisition and Pre-processing

**Figure 4:**
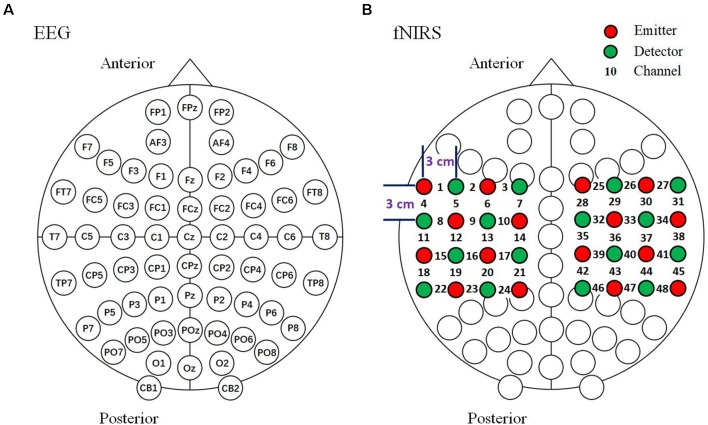
Arrangement of EEG and fNIRS channels. **(A)** EEG 64-channel arrangement based on the international 10–20 system. **(B)** Optode arrangement for fNIRS. The optodes were arranged above the bilateral parietal areas. Sixteen emitters and 16 detectors in the arrangement resulted in a total of 48 channels.

The current study used a 64-channel EEG and 48-channel fNIRS bimodal signals with simultaneous measurement (Figure 4). EEG signals were recorded with a Synamps2 EEG system (Neuroscan Synamps amplifier; Scan 4.5 Compumedics Corp., TX, USA) according to the international 10–20 system of electrode placement (Figure 4A). The reference electrode was placed on the left mastoid. Bipolar horizontal and vertical electrooculogram (EOG) derivations were recorded *via* two pairs of electrodes placed near the eyes. All electrode impedances were kept below 5 kΩ. EEG was recorded with a sampling frequency of 1,000 Hz and band-limited from 0.05 to 100 Hz, and a notch filter was used to suppress powerline interference. An independent component analysis (ICA) based on EEG and EOG was performed to remove eye movement and blink artifacts (see Ge et al., 2017 for details). During the data analysis, EEG signals were pre-processed with a bandpass filtered between 1 and 30 Hz. EEG data for each trial were corrected by subtracting the average of the data points between −700 ms and −500 ms.

The fNIRS signals recording were recorded with a LABNIRS system (Shimadzu Company Limited, Kyoto, Japan). The absorption of three wavelengths (780, 805 and 830 nm) of continuous near infrared light was measured with a sampling interval of 27 ms, then transformed into concentration changes of HbO, HbR and HbT by the modified Beer-Lambert law (Delpy et al., 1988). fNIRS optodes were positioned over the 64-channel EEG cap (Neuroscan, Charlotte, NC, USA) and the optodes and the electrodes were placed at intervals with a distance between the emitters and detectors of approximately 3 cm (Boas et al., 2004). We used a 48-channel system with 32 optodes (consisting of 16 emitters and 16 detectors) placed above the bilateral parietal areas. Channels 13 and 36 were set above the C3 and C4 areas, respectively (Figure 4B). In the current study, the locations of the optodes were measured using a 3D digitizer (FASTRAK; Polhemus, VT, USA). The fNIRS signals were pre-processed and bandpass filtered between 0.01 and 0.1 Hz. The fNIRS data for each trial were baseline-corrected by subtracting the average of the data points between −6.5 s to −0.5 s before observation period onset.

### Sensor-Level Analysis

The averaged event-related potential (ERP) and HbO waveforms of all channels for all participants were analyzed, respectively to investigate time series characteristics. In addition, a *t*-test of the HbO signal between the observation and baseline block of each channel was conducted from 1 to 5 s with 1-s steps to investigate the characteristics of fNIRS signals over time. Based on this analysis, the topographical maps of the *t*-test results of HbO concentration over the bilateral parietal areas were obtained using an interpolation method (using the griddata.m function of MATLAB 2013a, The MathWorks, Natick, MA, USA).

### Source-Level Analysis

For EEG source analysis, the grand average for 16 participants was first calculated with MATLAB. Next, the standardized low resolution electrical tomographic analysis (sLORETA)^1^, a functional imaging method based on electrophysiological and neuroanatomical constraints (Pascual-Marqui, 2002) was applied to the 16-participant grand average. sLORETA estimates the intracerebral electrical sources by calculating the scalp current source density (CSD) based on EEG signals (Pascual-Marqui et al., 2002). The sLORETA method has relatively good accuracy for source localization, even for deep sources (Keeser et al., 2011) and the average localization error is less than one grid unit (Pascual-Marqui, 2002). The sLORETA algorithm calculates the standardized CSD values at each of the 6,239 voxels of cortical gray matter, hippocampus, and amygdala at 5 mm spatial resolution in the digitized Montreal Neurological Institute (MNI) coordinates, corrected to Talairach coordinates (Talairach and Tournoux, 1988). This calculation of the standardized CSD is based upon a linear weighted sum of scalp electric potentials. The sLORETA algorithm solves the inverse problem by assuming that the neighboring neuronal sources have related dipole orientations and amplitudes (represented by adjacent voxels; Pascual-Marqui, 2002).

For fNIRS source analysis, the 3D coordinates of the anatomical markers (i.e., the nasion, inion, Cz, and left and right preauricular points) and fNIRS optodes (16 emitters, 16 detectors) were first digitized using the FASTRAK digitizer (Polhemus, Colchester, VT, USA). Meanwhile, the coordinates of the midpoints between each pair of the emitters and detectors were automatically calculated as the coordinates of the fNIRS channel. Second, the spatial registration was implemented between each fNIRS channel and *t*-test topographical maps. Third, the *t*-test topographical maps were superposed onto the surface of the MNI standard 3D head model using FUSION 3D imaging software (Shimadzu Co., Ltd.).

### Intention Classification Based on Complex Brain Network

For graph theoretical analysis, the complex brain network (Bullmore and Sporns, 2009) method is an emerging approach that can be applied to both anatomical and functional brain networks (Sporns, 2013). In the current study, we classified three different action intentions *via* complex brain networks based on EEG and fNIRS signals. First, we constructed two complex brain networks for EEG and fNIRS training datasets by treating EEG and fNIRS channels as nodes separately and determining their connections according to the Pearson’s correlation coefficient between each pair of channels. Second, five nodal features (i.e., nodal network properties), including the degree (the number of neighbors connecting to a node), clustering coefficient (the degree to which nodes in the network tend to cluster together), betweenness centrality (the proportion of the shortest paths pass through a node), eigenvector centrality (the degree of a node correlates with other nodes that are themselves central within the network) and local efficiency (how well information is exchanged by a node’s neighbors when it is removed) were calculated for EEG and fNIRS networks separately, according to the method reported in previous studies (Santiago et al., 2016; Fang et al., 2017; Zhao et al., 2017). Third, we combined the nodal features of EEG and fNIRS networks together, and selected the features based on the Relief-F algorithm (Kononenko, 1994), scoring the features based on the identification of feature value differences between nearest neighbor instance pairs using the relieff.m function of MATLAB with the nearest neighbors *k* = 10. Fourth, we used the programming library LIBSVM (C-supporting vector classification; Chang and Lin, 2011) for support vector machine (Yu et al., 2006) as the classifier. The grid search algorithm was employed to find the optimal values of the kernel parameter *γ* and penalty factor *C* (Ge et al., 2014). Finally, the classification accuracy was obtained using the averaged accuracies of 10 times 10-fold cross-validation (25 and three trials for training and testing, respectively).

## Results

**Figure 5:**
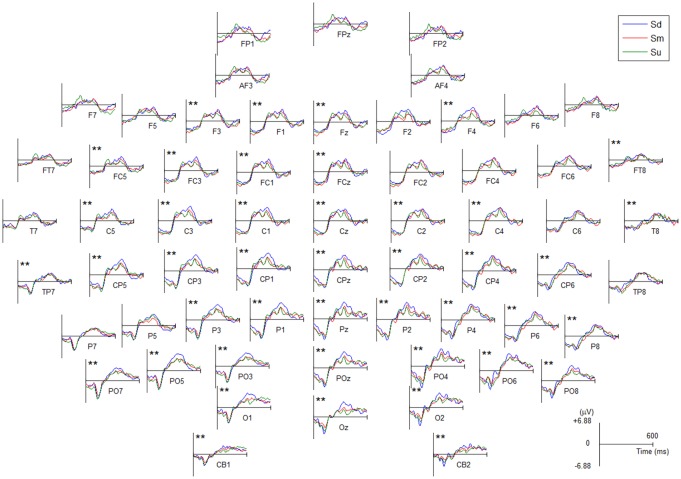
The averaged event-related potential (ERP) waveforms of all EEG channels for Sd, Sm and Su intentions for all participants. All channels have a negative amplitude at the 0 point because they were affected by the visual evoked potential induced by the −500 ms cue stimulus. Channel marked with two asterisks indicates a statistically significant difference in the amplitude (*P* < 0.001).

The averaged ERP waveforms of all channels for Sd, Sm and Su intentions for all participants are shown in Figure 5 and Supplementary Figure S1. Statistical analysis was performed with one-way analysis of variance (ANOVA) and a Bonferroni test for the multiple comparisons procedure was performed as a *post hoc* analysis (IBM SPSS version 21.0). The statistical results revealed a statistically significant difference in the mean amplitude of ERPs during 350–400 ms for the three intentions at 43 channels (see channels marked with two asterisks in Figure 5, among these 43 channels the lowest *F*_(2,150)_ = 50.7, *P* < 0.001). Moreover, the *post hoc* tests showed that among these 43 channels, Sd had a significantly greater amplitude than Sm and Su (highest *P* < 0.001, Bonferroni-corrected, respectively), while Sm had a significantly greater amplitude than Su (highest *P* < 0.001, Bonferroni-corrected).

**Figure 6:**
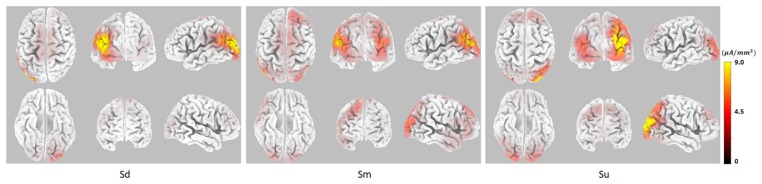
The EEG source analysis results between 350–400 ms for Sd, Sm and Su intentions for all participants.

EEG source analysis was implemented for the three intentions between 350–400 ms, revealing that, stable and identical activation regions were found during 350–400 ms, the Sd task mainly induced activation in the left hemisphere, including the middle occipital gyrus (MOG; BA18, 19), superior occipital gyrus (SOG; BA19), middle temporal gyrus (MTG; BA19, 39), superior temporal gyrus (STG; BA39), and angular gyrus (AG; BA39). The Sm task also induced activation that was mainly located in the left hemisphere, including the same activation areas, although this activation was weaker. In contrast, Su mainly induced right hemisphere activation, including the MOG (BA19), SOG (BA19), cuneus (BA19), MTG (BA19, 39), AG (BA39) and SPL (BA 7). The source analysis results between 350–400 ms are shown in Figure 6.

**Figure 7:**
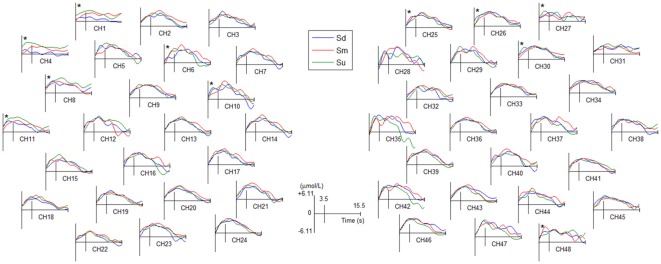
The averaged HbO waveforms of all fNIRS channels for Sd, Sm and Su intentions for all participants. All channels have a positive amplitude at the 0 point because they were affected by the visual evoked potential induced by the −500 ms cue stimulus. Channel marked with an asterisk indicates a statistically significant difference in the amplitude (*P* < 0.05).

The grand averaged HbO waveforms of fNIRS for three intentions are shown in Figure 7 and Supplementary Figure S2. The same ANOVA process used for grand averaged ERPs, as described in the previous paragraph, was used to analyze the difference of HbO waveforms among three intentions. The ANOVA statistical results revealed a statistically significant difference in the mean amplitude of HbO during 0–3.5 s for the three intentions at 11 channels (see channels marked with an asterisk in Figure 7, among these 11 channels the lowest *F*_(2,387)_ = 16.5, *P* < 0.05). Moreover, the *post hoc* tests showed that there are statistically significant differences between pairwise contrasts of the three conditions among these 11 channels (highest *P* < 0.05, Bonferroni-corrected). However, the differences were not identical in the three conditions among these 11 channels.

**Figure 8:**
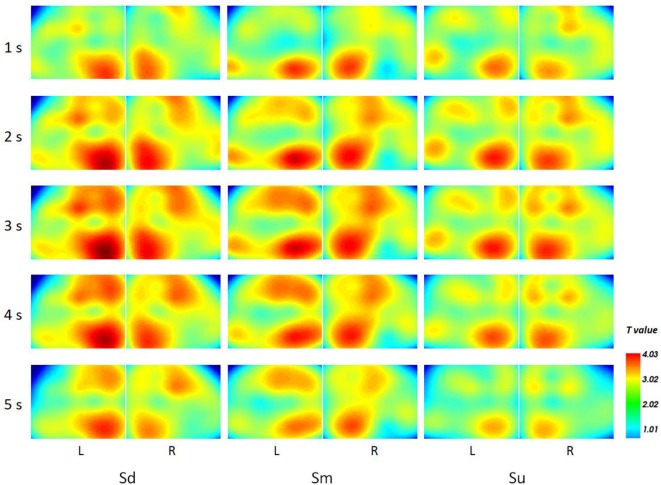
The topographical maps of *t*-test results of the HbO signal between the observation and baseline block of each channel from 1 to 5 s.

**Figure 9:**
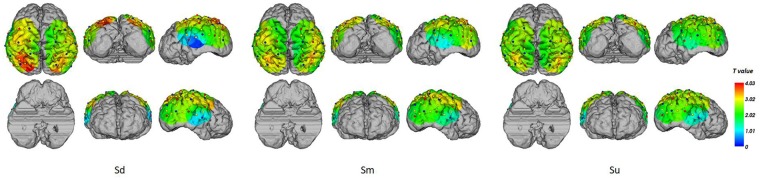
fNIRS source analysis results at 3 s for Sd, Sm and Su intentions for all participants.

Topographical maps of *t*-test results of the HbO signal between the observation and baseline block of each channel from 1 to 5 s for all participants are shown in Figure 8. A peak value can be clearly seen at 3 s. For this reason, we implemented fNIRS source analysis at 3 s. The results are shown in Figure 9. The results in Figure 9 indicate strong activations in the bilateral PMC, IFG, and TPJ for all three conditions, while activation in the left hemisphere was stronger than that in the right hemisphere. The *t*-values of the topographical map revealed that activation intensity exhibited a pattern of Sd > Sm > Su.

**Figure 10:**
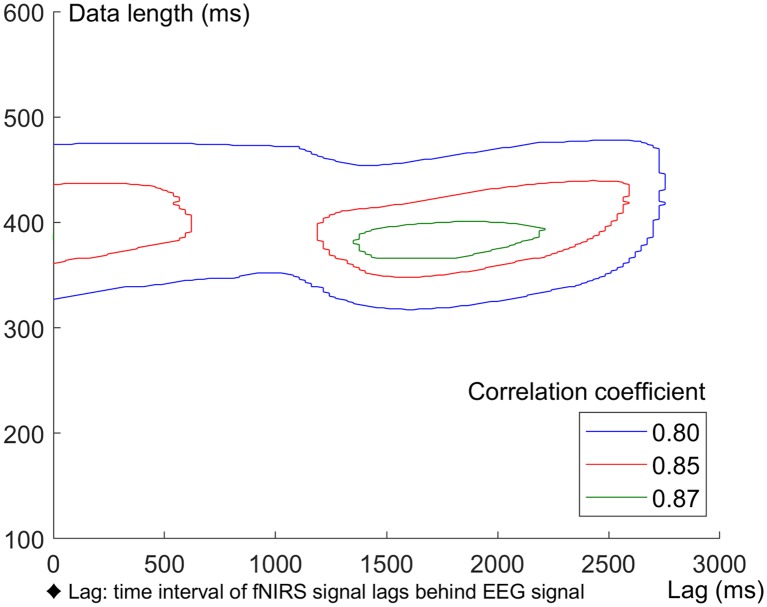
Correlation between ERP and HbO data under Sd, Sm and Su conditions. Areas with correlation coefficients greater than 0.8 for all the three conditions were plotted with contour lines.

In this study, we used a cross-correlation calculation method (Pfurtscheller et al., 2012; Sood et al., 2016) to compute the correlation between grand averaged ERP and HbO waveforms. For 0–X ms EEG data [X∈(100 600) ms, in steps of 1 ms], an equal length fNIRS data was selected and lagged Y ms behind EEG [Y∈(0 3,000) ms, in steps of 27 ms]. The correlation coefficients between ERP and HbO data for three different conditions (Sd, Sm and Su) were calculated, corresponding to every combination of X and Y. Areas with correlation coefficients greater than 0.8 for all three conditions (Sd, Sm and Su) were plotted with contour lines in Figure 10. The correlation analysis indicated a strong correlation between EEG and fNIRS when the length of the EEG and fNIRS recording was approximately 385 ms, and the fNIRS signal was around 1700 ms behind the EEG signal.

**Figure 11:**
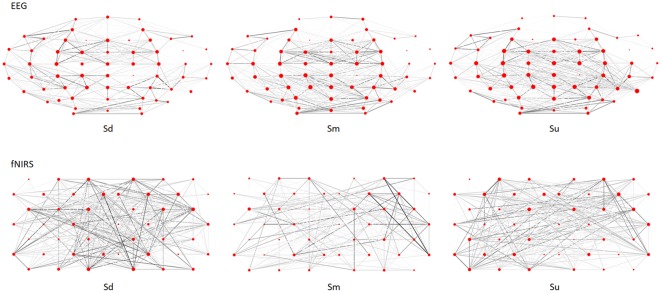
Complex brain networks of EEG and fNIRS datasets for participant No. 10.

The complex brain networks for the 0–3.5 s observation period of the EEG and fNIRS grand averaged datasets were calculated, respectively by treating channels as nodes, and determining the connections according to the Pearson’s correlation coefficient between each pair of channels, and normalizing these values to Fisher’s Z-values. A typical example of complex brain networks in the EEG and fNIRS datasets (participant No. 10) is shown in Figure 11. In this figure, a clear distinction can be seen between the complex networks of the three intentions.

The averaged classification results for three intentions of each participant based on the five features of complex brain networks calculated from EEG, fNIRS and EEG-fNIRS signals are shown in Table 1. The ANOVA results revealed a strong significant difference between three action observation tasks (*F*_(2,45)_ = 39.12, *P* < 0.001). In addition, EEG-fNIRS and EEG had significantly higher accuracy than fNIRS (*P* < 0.001, Bonferroni-corrected, respectively). Although the accuracy of EEG-fNIRS was higher than EEG, there was no significant difference between this two modes (*P* = 0.28, Bonferroni-corrected).

**Table 1:** The classification results for three intentions of each participant based on the five features of complex brain networks calculated from electroencephalography (EEG), functional near-infrared spectroscopy (fNIRS) and EEG-fNIRS signals.

Accuracy (%)	S1	S2	S3	S4	S5	S6	S7	S8	S9	S10	S11	S12	S13	S14	S15	S16	Avg	SD
EEG	65.1	68.9	80.2	60.2	82.5	73.3	76.4	63.3	69.9	56.9	64.6	71.6	62.6	68.2	70.4	63.7	68.6	6.8
fNIRS	56.3	61.4	51.8	53.2	55.2	57.2	50.1	48.0	55.1	59.0	54.7	70.1	50.4	37.7	44.3	38.9	52.7	7.9
EEG-fNIRS	73.4	70.2	79.5	65.9	80.1	71.5	81.2	74.3	75.1	73.5	67.1	73.0	68.3	69.4	68.9	72.3	72.7	4.4

*Avg: average; SD: standard deviation*.

**Figure 12:**
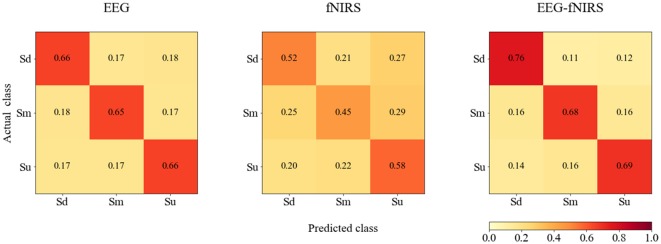
The averaged confusion matrices for EEG, fNIRS and EEG-fNIRS classifications for all participants.

The averaged confusion matrices for EEG, fNIRS and EEG-fNIRS classifications for all participants are shown in Figure 12.

## Discussion

In the current study, we found particularly strong activation in the AG, which is the main part of the IPL. This finding is consistent with previous human neuroimaging studies identifying the IPL as a key region for the coding of intention. This structure is part of a frontoparietal system that links observed and stored actions through direct matching (Rizzolatti et al., 2014) in terms of the actions themselves (Iacoboni, 2009) or goals (Hamilton, 2015). In addition, Reader et al.’s (2018) findings indicated that the IPL plays a role in kinematic processing of actions, regardless of whether they are meaningful or meaningless, which provides direct support for the current results.

The SPL has previously been reported to be activated during both action observation and execution (Rizzolatti et al., 2014) and is considered to play a critical role in mental rotation and spatial transformation (Buneo and Andersen, 2006; Lamm et al., 2007). Oh et al.’s (2019) model indicated that the SPL may be involved in visuospatial transformation, allowing the MNS to observe and imitate actions independently of demonstrator-imitator spatial relationships.

In accordance with Jelsone-Swain et al.’s (2015) findings, we observed activity in the MTG for all three task conditions. Accordingly, Kilner identified the MTG as the primary region bridging the two neural pathways involved specifically in action understanding (Kilner, 2011).

The current results revealed activation in primary visual regions including the MOG, SOG and cuneus for all three intention conditions. STG activation was observed in both the Sd and Sm tasks. The STG is a higher-order visual region and has been reported to play key roles in the identification of goal-directed movements (Schultz et al., 2004) and observation of biological motion (Matthys et al., 2009).

Previous studies using similar tasks reported that the mirror function of the brain is increased when the observed actions are familiar and have clear intentions (as in the “grasping a cup for drinking” and “grasping a cup for moving” conditions). In contrast, in the “touching a cup without clear intention” condition, the mirror function of the brain would be expected to be suppressed because the perceived action is outside the observer’s repertoire of familiar movements, which induces a lower level of activity (de Lange et al., 2008; Ge et al., 2017; Zhang et al., 2018). This tendency was observed in the current study, indicating that, during familiar action observation (Sd and Sm tasks), the processing of motor information leading to goal understanding (direct-marching process) requires more effort because of the complexity of the observed grip and its relationship with the object. However, such a process does not occur in the case of unfamiliar actions (Su), in which a less direct matching process is implemented.

Several previous studies concluded that the MNS network is active during the visual processing of others’ actions (how and what), while the ToM network is additionally recruited to process their intentions (why; de Lange et al., 2008; Spunt et al., 2010). In a recent review, Eren proposed that MNS and ToM functions are more complex than traditionally thought, with interrelated rather than independent functions (Eren, 2009). Based on Rizzolatti et al.’s (2014) definition, understanding the goal of a motor act performed by another individual involves two levels of insight: first, understanding of “what” the other is doing (e.g., grasping a cup); second, the understanding of “why” the other is doing it, which is considered the action overarching intention (e.g., grasping a cup for drinking). Previous studies have indicated that in low-level action understanding, the MNS obtains direct awareness of the goal of an action by recognizing what an action is and how it is being performed, particularly for familiar or frequently executed actions, whereas at a higher level of action understanding, the MNS might respond to why an action is being performed (Thioux et al., 2008; van Overwalle and Baetens, 2009). Many previous studies of action understanding suggested that the left hemisphere MNS is primarily related to the encoding of the motor act itself (what). In contrast, the right hemisphere MNS may be involved in the process of understanding the intentions underlying the actions (why; Ortigue et al., 2010; Ge et al., 2017). Some studies have proposed that, in the absence of context information or when observing unusual actions, the ToM network might be particularly strongly recruited to supplement insufficient information by inferring others’ mental states (de Lange et al., 2008; Spunt et al., 2011). In the current study, we found clear activation shifts from the left to the right hemisphere when changing from observing familiar actions with clear intentions (Sd and Sm tasks) to observing unfamiliar actions without a clear intention (Su task). This finding is in accord with previous studies by Ortigue et al. (2010), Ge et al. (2017) and Zhang et al. (2018). We assumed that when an action was more familiar or clear, direct-matching processing in the left hemisphere would be more strongly engaged. In contrast, we assumed that a more novel or ambiguous action would engage more inferential or mentalizing processes in the right hemisphere. The current results support the notion that observing the actions of people with clear intentions recruits the MNS, enabling an immediate understanding of the observed acts and of the agent’s intentions. However, when actions without clear intentions are observed, inferential or mentalizing processes based on the ToM appear to be engaged (Ge et al., 2017). Thus, our findings indicate that information processing during action observation is a complex process that cannot be attributed to a single neuronal mechanism.

Bimodal EEG-fNIRS measurement provides an approach to investigate brain activation with different spatial and temporal resolutions. EEG measures functional brain activity directly by detecting variations in electrical activity, which is a rapidly changing signal. In contrast, fNIRS measures functional brain activity indirectly *via* changes in the concentration of oxygenated and deoxygenated hemoglobin, which is a slowly changing signal. Previous ERP studies reported early mirror neuron activation of the P1, N170 and N400 components (Mohring et al., 2014), while the ToM or mentalizing network exhibited activation of the late negative ERP component at approximately 300–1,100 ms (Beudt and Jacobsen, 2015). The ERP waveforms revealed that mirror neuron ERP components oscillated between positive and negative components in a short time period, while the ToM or mentalizing network activation exhibited a long-term stable ERP component. Thus, the EEG source analysis in the current study may have reflected rapidly changing mirror neuron activation, while fNIRS source analysis may have reflected slowly changing activation of the ToM or mentalizing network. Because EEG and fNIRS have different imaging mechanisms and measure different physiological signals, the bimodal EEG-fNIRS measurement used in the current study may provide more complete biological information with different spatial and temporal resolutions (Putze et al., 2014). Thus, this combined method could enable more comprehensive knowledge of action observation and understanding.

In addition, bimodal EEG-fNIRS measurement can quantitatively study the NVC (Govindan et al., 2016), reflecting the mechanism linking transient neural activity to subsequent changes in CBF. A high temporal and spatial correlation exists between neuronal activity and CBF [i.e., brain regions with high activity exhibit an accompanying increase in the amount of blood flow (Hendrikx et al., 2019)]. In the current study, we also investigated the NVC between EEG and fNIRS signals and found a high correlation between these signals. This result was consistent with previous reports (Girouard and Iadecola, 2006; Govindan et al., 2016), indicating that neural activity is closely related to CBF. The correlation analysis between the grand averaged ERPs and HbO waveforms revealed a high correlation between EEG and fNIRS when the EEG and fNIRS length was approximately 385 ms (starting from 0 ms) and the fNIRS signal lagged approximately 1,700 ms behind the EEG signal. These results demonstrate the lag of the fNIRS signal behind the EEG signal and help to elucidate the temporal features of NVC corresponding to action intention understanding.

As we discussed in the preceding three paragraphs, the findings of previous studies and the current study indicated distinct differences in the timing and location of activation in response to different types of action intention observation. Such temporal and spatial dynamics of cortical activity can cause different topological characteristics of complex brain networks corresponding to observations of different types of action intentions (e.g., Figure 11), enabling the possibility of intention classification. Specifically, the current results indicated that three types of complex brain networks exist, each corresponding to the observation of different action intentions. Because different action intention observations cause differences in cortical activation, the topological characteristics of the three complex brain networks appear to be different. In the current study, we used complex brain networks to classify different types of action observations based on bimodal EEG-fNIRS signals. Table 1 reveals that the classification accuracy of EEG-fNIRS was 72.7%, which was higher than that of EEG or fNIRS alone. In addition, single-mode EEG achieved better performance than fNIRS (68.6% vs. 52.7%). This finding indicated that EEG provided more distinguishable complex network features than fNIRS for some participants. In addition, the results revealed that the fNIRS accuracy was very low for some participants, which reduced the corresponding EEG-fNIRS accuracy compared with the single-mode EEG accuracy for those participants. This finding suggests the possibility that future studies may be able to improve feature extraction and selection for fNIRS signals. Despite these limitations, the current study demonstrated that complex brain networks based on bimodal EEG-fNIRS provide a powerful classification method for action observation classification, which may have useful applications in HMI.

In addition, according to Figure 12, bimodal EEG-fNIRS achieved better classification performance than single-mode EEG and fNIRS, while fNIRS exhibited the worst performance. For EEG, no classification difference was observed among the three classes. For fNIRS, the Sm class was most often misclassified. For EEG-fNIRS, the Sd class exhibited higher classification accuracy than the Sm and Su classes. These results suggest that although single-mode fNIRS achieved intermediate classification performance, bimodal EEG-fNIRS signals could further improve classification performance. These findings suggest the possibility that further studies examining feature extraction and selection for fNIRS signals may be able to improve the classification performance of bimodal fNIRS and EEG-fNIRS systems.

## Conclusion

To investigate the neural basis of action observation and understanding, we used bimodal EEG-fNIRS measurement to investigate the sensor- and source-level activations for action observation. The results indicated that information processing during action observation is a complex process involving the MNS and ToM networks. In addition, we tested a proposed method using complex brain networks to classify the brain activations for different action observation tasks. By combining the features of EEG and fNIRS complex brain networks, the method achieved a satisfactory classification accuracy (72.7%), demonstrating the possibility of encoding action observation and understanding.

## Ethics Statement

All participants provided written informed consent before enrolment in the study, which was approved by The Ethics Committee of Affiliated Zhongda Hospital, Southeast University (2016ZDSYLL002.0 and 2016ZDSYLL002-Y01). All participants gave written informed consent in accordance with the Declaration of Helsinki. Each participant received 200 Chinese Yuan for participating after the experiment.

## Author Contributions

SG conducted the experiments, data analysis and wrote the manuscript. PW, HL and PL conducted the experiments and data analysis. JG, RW and KI developed the experimental designs. QZ and WZ designed the research.

## Conflict of Interest

The authors declare that the research was conducted in the absence of any commercial or financial relationships that could be construed as a potential conflict of interest. The handling editor is currently co-organizing a Research Topic with one of the reviewers ZY, and confirms the absence of any other collaboration.

## Footnotes

^1^http://www.uzh.ch/keyinst/loreta.htm
