# Supplementary material for: Neural Activity and Decoding of Action Observation Using Combined EEG and fNIRS Measurement
Source: Front Hum Neurosci. 2019 Oct 15;13:357. doi: 10.3389/fnhum.2019.00357 (PMC6803538; doi:10.3389/fnhum.2019.00357)
Supplement: Supplementary file 1 [file Data_Sheet_1.pdf]

Supplementary Material

Neural Activity and Decoding of Action Observation Using Combined EEG and fNIRS Measurement

Sheng Ge, Peng Wang, Hui Liu, Pan Lin, Junfeng Gao, Ruimin Wang, Keiji Iramina, Quan Zhang, Wenming Zheng*

*** Correspondence:** Wenming Zheng: wenming_zheng@seu.edu.cn

1. Supplementary Figures

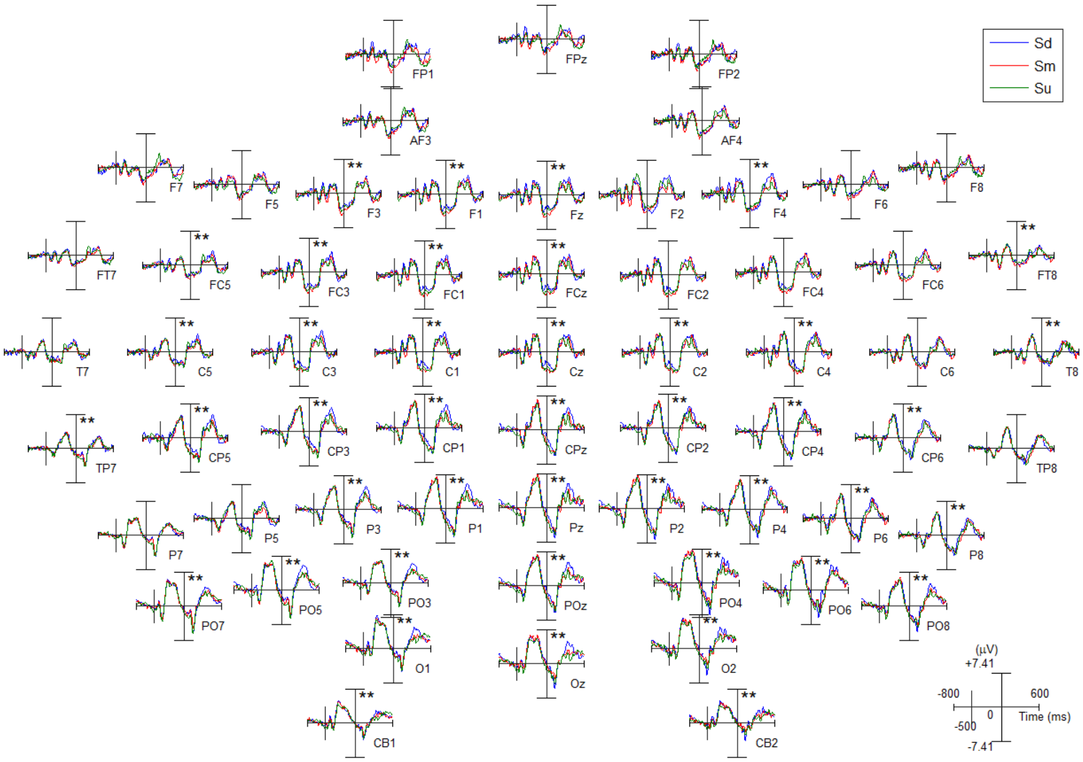

Supplementary Figure 1. The averaged ERP waveforms of all EEG channels for the Sd, Sm, and Su intentions for all participants.

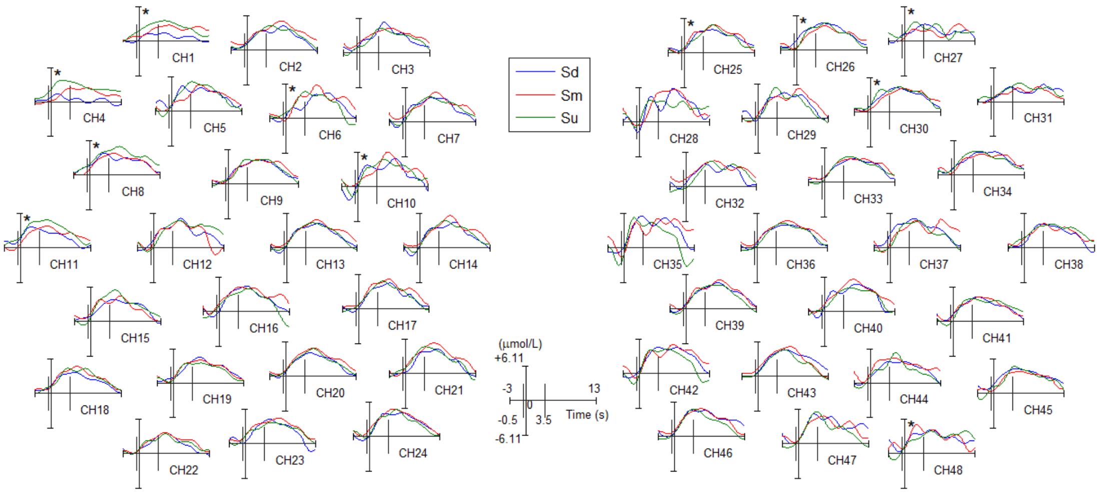

Supplementary Figure 2. The averaged HbO waveforms of all fNIRS channels for the Sd, Sm, and Su intentions for all participants.
